# Catheter-Related Bladder Discomfort: Insights Into Pathophysiology, Clinical Impact, and Management

**DOI:** 10.7759/cureus.81322

**Published:** 2025-03-27

**Authors:** Titos Markopoulos, Stamatios Katsimperis, Lazaros Lazarou, Lazaros Tzelves, Iraklis Mitsogiannis, Athanasios Papatsoris, Andreas Skolarikos, Ioannis Varkarakis

**Affiliations:** 1 Second Department of Urology, National and Kapodistrian University of Athens, Sismanogleio General Hospital, Athens, GRC; 2 Second Department of Urology, National and Kapodistrian University of Athens, Sismanoglio General Hospital, Athens, GRC

**Keywords:** bladder pain, bladder spasms, catheter discomfort, catheter-related bladder discomfort, transurethral surgery

## Abstract

Catheter-related bladder discomfort (CRBD) is a frequent postoperative complication that significantly impacts the quality of recovery in patients undergoing transurethral surgeries. It manifests as suprapubic pain, an intense urge to void, and bladder spasms due to muscarinic receptor activation. This narrative review provides a comprehensive analysis of CRBD, with a particular focus on bladder spasms in patients treated with transurethral resection of bladder tumors (TURBT) or the prostate (TURP). The review explores the underlying pathophysiology, clinical implications, and evidence-based management strategies, including pharmacological interventions such as antimuscarinics and nerve blocks, as well as non-pharmacological measures.

## Introduction and background

Catheter-related bladder discomfort (CRBD) is a distressing condition that primarily arises from the use of indwelling urinary catheters in postoperative patients. The symptoms, including suprapubic pain, bladder spasms, and a constant urge to void, mimic those of overactive bladder (OAB) but are unique in their etiology [1-4]. CRBD is particularly prevalent among patients undergoing transurethral surgeries such as transurethral resection of bladder tumors (TURBT) and the prostate (TURP), where catheterization is essential for managing postoperative bleeding and irrigation [5, 6].

The incidence of CRBD is alarmingly high, ranging from 47% to 90%, depending on the type of surgery and patient population [7-11]. This condition not only prolongs recovery but also significantly impacts patient satisfaction, increases healthcare costs, and heightens the risk of postoperative complications [7, 12]. This review aims to delve into the mechanisms of CRBD, elucidate its impact on patients undergoing transurethral surgeries, and discuss effective management strategies. Special attention is given to bladder spasms, a hallmark feature of CRBD, which exacerbate patient discomfort and challenge clinical management.

## Review

Pathophysiology of CRBD and bladder spasms

The mechanism of CRBD is similar to that of overactive bladder, which is caused by involuntary contractions of the bladder mediated by muscarinic receptors [13]. CRBD is primarily mediated by the activation of muscarinic receptors in the detrusor muscle of the bladder, which are heterogeneously distributed, with M2 receptors being the dominant ones and M3 receptors less prominent. Although M2 receptors predominate in the bladder, the subtype M3 receptors are primarily responsible for bladder contraction [14]. These receptors, when stimulated, induce involuntary bladder contractions, commonly known as bladder spasms [3, 14, 15].

The presence of an indwelling catheter and bladder irrigation exacerbate this response by irritating the urothelium and stimulating afferent nerve fibers, leading to heightened acetylcholine release and detrusor overactivity. When a catheter is connected to a urine bag, it allows the bladder to empty; however, the catheter tip can irritate the bladder wall, potentially leading to erosion. Contact between the tip of a catheter and the bladder trigone is particularly painful. The discomfort, often referred to as "catheter cramp," results from bladder and urethral spasms caused by the catheter irritating the bladder wall and trigone. The body reacts to the indwelling urinary catheter, which is a foreign body, and the balloon of the catheter tip adds pressure to the bladder neck and pelvic floor. Balloon size seems to play a major role. Another potential etiology is urethra stimulation. In a prospective study, Binhas et al. studied the role of the Foley catheter size as a predictive factor for bladder spasms, demonstrating that discomfort increases notably when the catheter size exceeds 18F, especially in men [6]. They attributed this finding to the anatomical difference in urethral length, as the male urethra is substantially longer than the female urethra. Despite its clinical significance, CRBD linked to urethral pain remains underexplored, underscoring the pressing need for more focused research in this area.

Another underlying mechanism of CBRD is the elevated concentration of prostaglandins in the urine. This increase is linked to mucosal injury caused by the urinary catheter, which initiates localized inflammation. Inflammation activates the cyclooxygenase pathway, resulting in the release of prostaglandins [16-19]. Medications with anti-inflammatory properties, such as paracetamol and ketorolac, are recognized as effective options for managing postoperative CBRD [16, 17].

Clinical impact on transurethral surgery patients

The clinical implications of CRBD in patients undergoing TURBT and TURP are profound. Bladder spasms, the hallmark of CRBD, not only cause significant discomfort but also interfere with surgical outcomes. For instance, in TURBT patients, bladder spasms can dislodge hemostatic clots, leading to secondary hemorrhage and delayed wound healing. In TURP patients, severe spasms increase the risk of catheter dislodgement, potentially causing urethral trauma and urinary retention.

CRBD also affects postoperative recovery timelines. Patients experiencing moderate to severe CRBD often require higher doses of analgesics and sedatives, prolonging their hospital stays and increasing the likelihood of side effects such as respiratory depression and constipation [3, 7, 12]. Moreover, the psychological impact of CRBD is substantial. Patients report increased anxiety and dissatisfaction with their surgical experience, which can affect their overall perception of care quality [20, 21].

The economic burden of CRBD is another critical consideration. Studies estimate that unresolved CRBD can increase healthcare costs due to extended hospital stays, additional medications, and increased nursing care [22]. This underscores the importance of implementing effective preventative and therapeutic strategies to mitigate the impact of CRBD on both patients and healthcare systems.

Management approaches

Pharmacological Strategies

Antimuscarinic Agents: Antimuscarinic drugs such as oxybutynin, tolterodine, and solifenacin are first-line treatments for CRBD. These agents work by blocking M3 receptors in the bladder, reducing detrusor overactivity and alleviating spasms. Additionally, they increase bladder capacity by inhibiting the bladder-afferent mechanism during bladder filling [23]. Although effective, antimuscarinics are known to cause side effects, including xerostomia, constipation, and potential cognitive impairment, particularly in older adults [24]. Solifenacin has demonstrated superior efficacy and tolerability compared to older agents, making it a preferred choice in postoperative settings. Its efficacy has been demonstrated in prospective studies [25, 26]. Zhang et al. divided patients who underwent TURBT into two groups and compared their symptoms [26]. In this study, 58 patients were administered 5 mg of solifenacin, while another 58 received a placebo, with the first dose given 6 hours before surgery. CRBD was evaluated at intervals of 6, 12, 24, 48, and 72 hours following surgery. The treatment continued for two weeks postoperatively, and symptom scores for overactive bladder (OAB) were compared between the two groups. Patients in the solifenacin group experienced a significant decrease in the incidence and severity of CRBD (P<0.05) and a notable improvement in OAB symptoms (P<0.001) compared to those in the placebo group.

Lidocaine, though not classified as an antimuscarinic agent, has demonstrated significant antimuscarinic and anti-inflammatory properties, making it a valuable option for the treatment of CRBD. Previous research has demonstrated that lidocaine effectively inhibits muscarinic receptor activity and has the ability to suppress inflammatory responses mediated by immune cells [27]. A randomized controlled trial conducted by Kim et al. investigated whether intravenous lidocaine administration reduces postoperative CRBD in male patients undergoing TURBT [28]. Patients were randomly assigned to receive either intravenous lidocaine (administered as a 1.5 mg/kg bolus followed by a continuous infusion of 2 mg/kg/h during the surgery and for 1 hour postoperatively) or a placebo (normal saline). The study identified three key outcomes. Firstly, intravenous lidocaine effectively reduced the occurrence of moderate-to-severe CRBD in male patients undergoing TURBT who required large-diameter urinary catheters. Secondly, the use of intravenous lidocaine significantly enhanced patient satisfaction by preventing CRBD. Lastly, no substantial side effects associated with lidocaine were detected [28]. The authors noted that the neurologic and cardiac side effects of lidocaine typically occur when plasma concentrations exceed 5 μg/mL. However, intravenous lidocaine administered at standard doses (1-2 mg/kg as an initial bolus, followed by a continuous infusion of 0.5-3 mg/kg/h) generally results in plasma concentrations below this threshold. As a result, no side effects were observed in their study.

Gabapentinoids: Gabapentin and pregabalin, known for their neuromodulatory effects, are increasingly being used to manage CRBD. These agents reduce neuronal excitability, providing relief from both sensory discomfort and spasms. Preoperative administration of gabapentinoids has shown a significant reduction in CRBD incidence [2, 7, 15, 29].

Gabapentin, a structural analog of gamma-aminobutyric acid (GABA), is widely recognized for its antiepileptic, antinociceptive, and analgesic properties [30]. The drug functions as a ligand for the α2δ subunit of voltage-sensitive calcium channels, inhibiting the activation of afferent C-fibers and αδ fibers [31]. This inhibitory action helps reduce peripheral sensitization, a mechanism implicated in conditions like overactive bladder (OAB), sensory urgency, and urge incontinence [32]. Gabapentin is believed to modulate detrusor overactivity by regulating afferent signals from the bladder and decreasing the excitability of the sacral reflex center [33]. Clinically, gabapentin has been used in doses of up to 3000 mg for refractory cases of OAB, particularly when conventional antimuscarinic therapies fail [32]. It has demonstrated efficacy in alleviating OAB symptoms, improving urodynamic parameters, and managing neurogenic detrusor overactivity. Furthermore, its role in reducing bladder afferent nerve function supports its potential utility in treating CRBD.

Pregabalin is a medication primarily used to manage neuropathic pain, epilepsy, and generalized anxiety disorder. It is also employed for conditions like fibromyalgia and overactive bladder symptoms. Classified as an anticonvulsant and analgesic, pregabalin works by modulating nerve signaling rather than directly interacting with neurotransmitter systems like GABA, despite being a structural analog of GABA [34]. Instead, it targets the alpha2-delta subunit of voltage-gated calcium channels, where its strong affinity reduces calcium entry into nerve terminals during depolarization [35]. This reduction decreases the release of excitatory neurotransmitters, which is believed to underlie its analgesic properties [36]. In the context of bladder function, pregabalin may help reduce detrusor contractions and smooth muscle activity by limiting the peripheral release of excitatory signals. This action can increase bladder capacity by lengthening the time between episodes of urgency. Pregabalin is also more effective at lower doses compared to gabapentin, due to its enhanced bioavailability, faster absorption, and greater potency [34].

Srivastava et al. evaluated pregabalin’s efficacy in the prevention of postoperative CRBD in patients undergoing spine operations [29]. Sixty patients undergoing elective spine surgery and requiring urinary bladder catheterization were included in this study. The patients in the pregabalin group received 150 mg of pregabalin orally 1 hour before induction of anesthesia and the patients in the control group received placebo. CRBD incidence was significantly lower in the pregabalin group compared with the placebo group at all time intervals (P<0.05) [29]. The pregabalin group experienced a decrease in CRBD severity and postoperative fentanyl use compared to the control group, although this was accompanied by increased sedation.

Analgesics: Analgesics play a significant role in the management of CRBD. These medications primarily aim to alleviate the discomfort and pain associated with CRBD and improve patient outcomes. Nonsteroidal anti-inflammatory drugs (NSAIDs), such as ketorolac, reduce inflammation by inhibiting the cyclooxygenase (COX) pathway, thereby decreasing the production of prostaglandins that sensitize afferent nerves in the bladder. Ketorolac has been utilized in urologic surgery and has demonstrated notable effectiveness in reducing bladder spasms [16, 37]. 

Paracetamol, while lacking significant anti-inflammatory effects, modulates central pain pathways and is commonly used for mild to moderate pain relief in CRBD. In a prospective study by Ergenoglu et al., the administration of a single-dose paracetamol intraoperatively was found to be effective in reducing the severity of CRBD and pain in patients undergoing percutaneous nephrolithotomy [17].

Adjunctive Therapies - Magnesium, Ketamine, and Dexmedetomidine: Magnesium sulfate, a smooth muscle relaxant, offers a promising adjunct to standard therapy. Magnesium plays a vital role in numerous physiological processes within the human body, including cell membrane signal transduction, protein synthesis, nerve and muscle transmission, neuromuscular conduction, blood pressure regulation, and glucose metabolism [38, 39]. Additionally, magnesium is pivotal in maintaining normal nerve and muscle function, as it regulates the transmission of electrical signals in neurons and supports muscle contraction and relaxation by facilitating the active transport of calcium ions across cell membranes [39]. Given these physiological functions, Park et al. conducted a randomized, double-blind, placebo-controlled study to evaluate whether intraoperative magnesium reduces moderate-to-severe CRBD in patients recovering from TURBT [40]. In this trial involving 120 patients, intravenous magnesium significantly alleviated discomfort, with a number needed to treat (NNT) of just 2, while also enhancing patient satisfaction, highlighting magnesium as a simple and cost-effective option for reducing bladder discomfort.

Ketamine is a dissociative anesthetic and analgesic medication that has been widely used by anesthesiologists. It was originally developed as an alternative to phencyclidine (PCP) and is valued for its ability to induce anesthesia and analgesia without significantly depressing respiratory or cardiovascular function. Its interaction with opioid, monoaminergic, and muscarinic receptors contributes to its analgesic and anesthetic effects [41]. Ketamine was incidentally found to be effective in managing CRBD when it was administered to treat postoperative shivering [42]. Building on this observation, the same team conducted a trial to evaluate ketamine's efficacy as a treatment for CRBD during the postoperative period. Their findings revealed that ketamine significantly reduces both the incidence and severity of postoperative CRBD [43]. Safavi et al. reported similar findings in their study, which evaluated the efficacy of various ketamine doses compared to placebo for the treatment of CRBD in patients undergoing endourological procedures such as TURBT, cystoscopy, bladder neck biopsy, lithotripsy, and double-J ureteric stent placement [44].

Dexmedetomidine, a selective α2-adrenergic agonist with sedative and analgesic properties, has also demonstrated efficacy in reducing catheter-related bladder discomfort (CRBD). In a randomized, placebo-controlled study involving patients undergoing transurethral resection of bladder tumors (TURB), intraoperative dexmedetomidine significantly decreased both the incidence and severity of CRBD during the early postoperative period. It also reduced intraoperative desflurane requirements and postoperative opioid use, supporting its role as a useful anesthetic adjuvant in this setting [5].

Pudendal Nerve Blocks (PNBs): Pudendal nerve blocks provide targeted relief by blocking afferent nerve signals from the bladder. Ultrasound-guided PNBs have shown significant efficacy in reducing CRBD severity and postoperative analgesic requirements.

Two recent studies have highlighted the efficacy of PNB in reducing CRBD and associated complications in patients undergoing urological surgeries. Xiaoqiang et al. conducted a randomized, double-blind, placebo-controlled trial involving 175 male patients undergoing lower urinary tract surgeries, including TURP and TURBT [45]. Their findings demonstrated that PNB significantly decreased the incidence and severity of CRBD while also alleviating postoperative pain and improving hemodynamic stability, with no reported complications.

Similarly, Wang et al. evaluated the effect of bilateral, ultrasound-guided PNB at the pudendal (Alcock’s) canal in 110 patients undergoing holmium laser enucleation of the prostate (HoLEP) [22]. The results revealed a substantial reduction in both bladder spasms and CRBD during the first 24 hours postoperatively, alongside decreased analgesic requirements and improved patient satisfaction. This study further emphasized the safety and efficacy of PNB, with no complications related to the procedure.

These findings underscore the potential of PNB as a safe, effective, and minimally invasive strategy for managing CRBD and improving postoperative outcomes in patients undergoing endourological procedures.

Non-pharmacological Strategies

Catheter Optimization: Optimizing catheter-related parameters has emerged as an effective non-pharmacological strategy to reduce CRBD. Zugail et al. investigated the impact of reducing the balloon volume in indwelling urinary catheters (IUCs) on CRBD severity [3]. Their prospective study, involving 49 patients, demonstrated that halving the balloon volume significantly alleviated CRBD symptoms and pain, as assessed by a visual analog scale and CRBD grading system. This simple and non-invasive adjustment improved patient comfort without compromising catheter functionality, highlighting its potential as a practical adjunct to pharmacological therapies.

In a related study, Zhu et al. examined the effect of different catheter fixation sites on catheter-associated lower urinary tract symptoms (CALUTS), a condition closely linked to CRBD [46]. Their analysis of 450 patients undergoing upper urinary tract surgeries compared three groups: thigh fixation, abdominal wall fixation, and no fixation. The results revealed that thigh fixation markedly reduced urgency, frequency, and discomfort compared to the other groups. Abdominal wall fixation also offered a moderate reduction in CALUTS, though it was less effective than thigh fixation. These findings suggest that refining catheter fixation techniques can further enhance patient comfort and minimize CRBD-related symptoms.

Complementary Therapies: Techniques like transcutaneous electrical nerve stimulation (TENS) and acupuncture have shown promise in alleviating CRBD symptoms. These non-invasive methods modulate sensory input and improve bladder function, providing an alternative for patients intolerant to pharmacological therapies.TENS therapy applies mild electrical impulses to nerve pathways, which may reduce afferent sensory input from the bladder and thereby minimize discomfort. Clinical studies have demonstrated that TENS, when applied at specific acupuncture points, significantly decreases CRBD symptoms and the need for additional pharmacological intervention. This technique, which is non-invasive and free of systemic side effects, holds promise as a complementary therapy for postoperative patients at high risk of CRBD. Liang et al. conducted a randomized, double-blind, placebo-controlled study to assess the efficacy of transcutaneous electrical acupoint stimulation (TEAS) in reducing CRBD in patients undergoing TURP [47]. The study involved 70 patients, with TEAS applied at specific acupoints 30 minutes before anesthesia. The results demonstrated that TEAS significantly reduced both the incidence and severity of CRBD at various postoperative time points. Additionally, TEAS decreased postoperative analgesic requirements and improved recovery quality as measured by QoR-40 (quality of recovery 40 items) scores [47]. This study highlighted TEAS as a non-invasive and effective complementary therapy for managing CRBD.

Exploring neuromodulation techniques, such as sacral nerve stimulation (SNS), may also offer new avenues for CRBD management. SNS involves the implantation of an electrode near the sacral nerves, which play a critical role in bladder control. This technique modulates abnormal detrusor activity by sending low-intensity electrical impulses to the sacral nerve roots (S3-S4), thereby reducing detrusor overactivity and bladder spasms. While traditionally used for refractory overactive bladder and neurogenic bladder dysfunction, SNS has shown promise in reducing urgency, frequency, and involuntary bladder contractions associated with catheterization. These findings indicate that SNS could be particularly beneficial for patients who experience severe CRBD unresponsive to conventional pharmacological treatments. Future research should aim to establish optimal programming parameters and assess long-term efficacy in post-surgical CRBD patients.

## Conclusions

The management of CRBD requires a comprehensive approach that addresses its multifactorial etiology. Pharmacological treatments, while effective, are often associated with side effects and variable efficacy. Non-pharmacological interventions, such as catheter optimization and neuromodulation techniques, offer valuable adjuncts that can enhance patient outcomes. The integration of complementary therapies into standard care protocols represents a promising frontier in CRBD management.

Future research should focus on the long-term outcomes of existing therapies and the development of innovative approaches that target the underlying mechanisms of CRBD. Interdisciplinary collaboration among urologists, anesthesiologists, and researchers will be pivotal in advancing the field.
